# A three-decade review of telemetry studies on vultures and condors

**DOI:** 10.1186/s40462-018-0133-5

**Published:** 2018-09-04

**Authors:** Pablo A. E. Alarcón, Sergio A. Lambertucci

**Affiliations:** 1 Grupo de Investigaciones en Biología de la Conservación, Laboratorio Ecotono, INIBIOMA (Universidad Nacional del Comahue-CONICET), Quintral 1250 (R8400FRF), Bariloche, Argentina; 2 Grupo de Ecología Cuantitativa, INIBIOMA (Universidad Nacional del Comahue-CONICET), Quintral 1250 (R8400FRF), Bariloche, Argentina

**Keywords:** Animal tracking, Avian scavenger, Movement ecology

## Abstract

Telemetry-based movement research has become central for learning about the behavior, ecology and conservation of wide-ranging species. Particularly, early telemetry studies were conducted on vultures and condors due to three main reasons: i) these birds capture the curiosity of humans, ii) their large body size allows researchers to deploy large telemetry units, and iii) they are of high conservation concern. This has resulted in a great number of scientific articles that remain scattered throughout the literature. To achieve a more cohesive view of vultures and condors movement behavior, we review all telemetry studies published up to 2017. We first present a descriptive summary of the technical and design characteristics of these studies (e.g. target species, tagging location, number of individuals tagged) and go on to discuss them under a common conceptual framework; the Movement Ecology Paradigm. The articles found (*N* = 97) were mainly published in the last decade and based on the tagging of individuals from 14 species (61% of the extant species) and 24 countries. Foraging was the most in-depth investigated movement phase (25 studies), with studies covering several species, using both phenomenological and mechanistic approaches and tackling the role of different drivers of movement. In contrast, commuting and natal dispersal phases were only superficially investigated (3 and 8 studies, respectively). Finally, studies dealing with the conservation and management also comprised a large portion of the reviewed articles (24 studies). Telemetry studies have revealed relevant details of vultures and condors movements, with highly accurate measurements of flight energetics and a better understanding of the morphological, physiological and context-dependent drivers that underlie the movement decisions of these birds. However, we also detected several information gaps. We expect this review helps researchers to focus their efforts and funds where more information is needed.

**Electronic supplementary material:**

The online version of this article (10.1186/s40462-018-0133-5) contains supplementary material, which is available to authorized users.

## Background

The movement of animals has attracted human’s attention for centuries, but recently there has been an increased interest that reflects in the scientific literature [1] most likely owing to two main reasons. Firstly, there exists a growing recognition that many questions in ecology and conservation biology cannot be properly answered if they are not posed in a spatial context where the consequences of movement can be evaluated [2–4]. Secondly, during the last decades a number of tools have become increasingly available to researchers that allow for the collection, storage, visualization and analysis of large volumes of fine-scale spatiotemporal data, including animal locations and biologically relevant data [5, 6]. These advances in the study of movement are now promoting an exciting and fast-moving science and providing a more integrated view of a key phenomenon for almost every ecological and evolutionary process [7]. Because of the rapid progress of this new discipline, there is a need for bringing together the large amounts of data that have become scattered throughout the literature in an effort to identify current strengths and weaknesses, especially in the case of poorly-understood and vulnerable species.

The new era of movement ecology has been driven by our interest in certain, often charismatic species. In this sense, vultures and condors ('vultures' from now on) have functioned as catalytic species owing to they are able to fly great distances over difficult-to-access habitats, a phenomenon that has aroused human’s curiosity and admiration since immemorial times and stimulated research on different aspects of their physiology, behavior, ecology [8]. Likewise, these large-bodied birds have served as models in the implementation of animal-attached devices which were initially large and heavy [6]. As a result, vulture movements can be now tracked in multiple environments and at resolutions that were hitherto unconceivable [9–11]. Likewise, sensors like heart-rate loggers and accelerometers are making possible to investigate the factors that underpin the movement decisions of these behaviorally complex birds; decisions which are made on the basis of much and varied information (e.g., physiological, weather, and social information) [12–14]. Finally, the fragile conservation status of many vulture species is yet another reason for us to understand their movement patterns. Vultures are among the vertebrate groups most affected by modern human societies, with some species reaching global population declines of up to 99% [15]. At present, 12 out of 23 vulture species worldwide face serious conservation crises putting a number of ecological, economic, sanitary and cultural services at risk [16–18]. Thus, the study of the movement ecology of vultures will mean a big step towards knowing their biology, but also towards the elaboration of well-informed conservation strategies.

In this work, we review how tracking technologies are being used by researchers and managers to gain insight into the behavior, ecology and the conservation of vultures. Our goal was twofold: i) to compile, synthesize and describe the existing literature and, ii) to bring these studies together under a common conceptual framework. For the latter purpose, we adopted the principles of the Movement Ecology Paradigm [7]. Within this paradigm, three main axes guide the study of movement. The first one consists of identifying ecologically relevant movement phases (e.g. foraging) in the life-track of focal individuals. The second axis consists of evaluating those movement phases from both phenomenological and mechanistic points of view; i.e. describing both movement patterns and underlying processes that create them. Finally, the third axis requires understanding the mechanistic bases of movement taking into account the interaction among four main components: the internal state of the focal individual, its motion and navigation capacities and external factors. Thus, we identified movement phases studied in vultures differentiating between phenomenological and mechanistic approaches and evaluated which, and to what extent, the above mechanistic components were addressed. Our work offers a broad overview of the current knowledge of the movement behavior of vultures, organized in a way as to identify the strengths and gaps in this knowledge as well as possible directions for future research. We expect this review will encourage researchers to investigate on the subjects where more information is needed and to locate the appropriate funds to do so.

## Methods

We performed a comprehensive electronic search in Scopus and Google Scholar to identify all available literature related to vulture movements up to December 2017. We used the terms ‘vulture’ or ‘condor’ or ‘bird scavenger’ or ‘avian scavenger’ in combination with either the term ‘tracking’, ‘telemetry’, ‘GPS’ or ‘movement’. In addition, we included any additional study cited in the references of the articles found via our electronic database search.

**Fig. 1:**
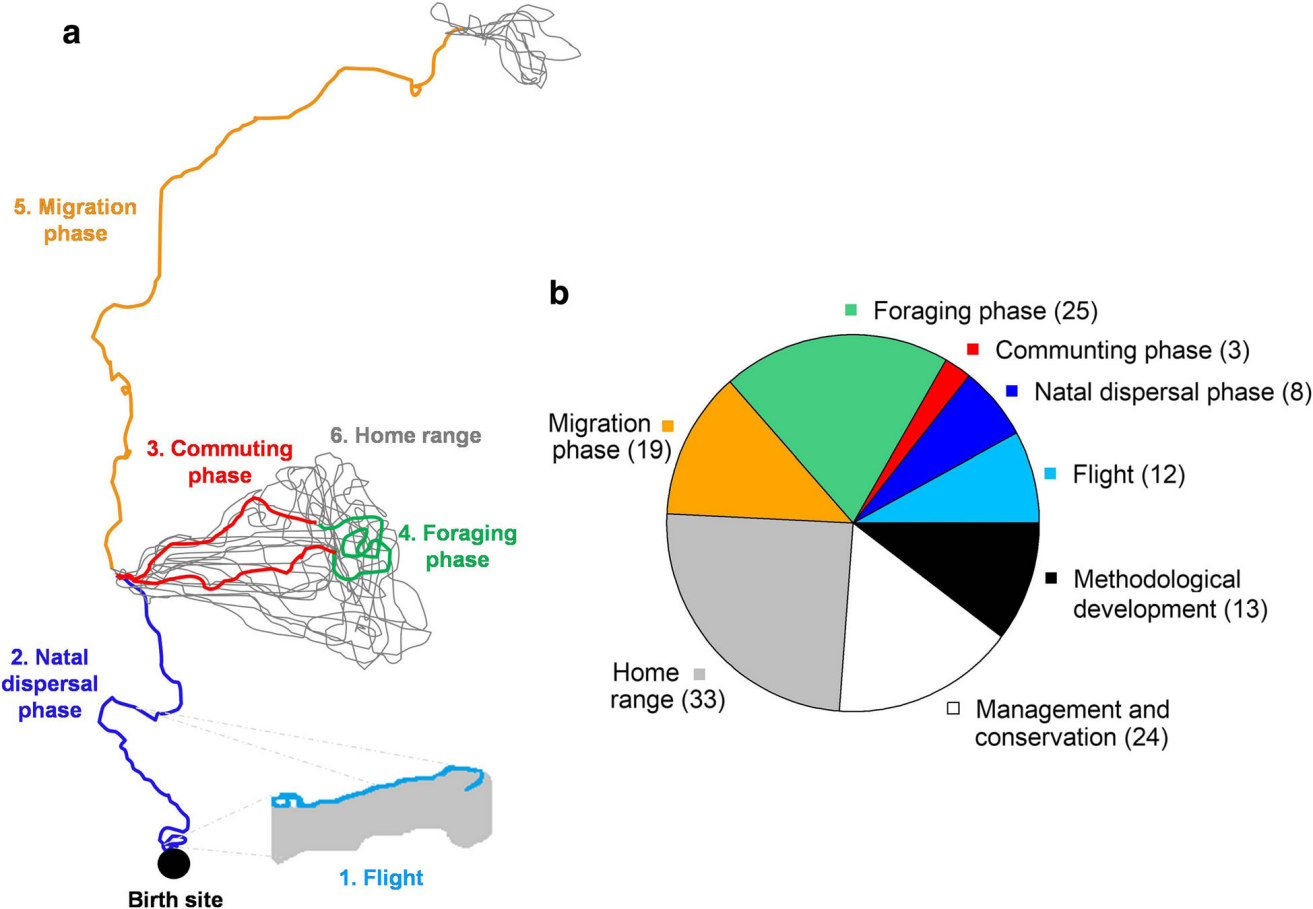
**a**. Schematic representation of a lifetime track involving all sections of movement addressed by telemetry studies conducted on vultures and condors. **b**. Frequency of studies for each studied category

From each scientific article we extracted information on both the publication (e.g. name and type of journal and year of publication) and the study itself (e.g. purpose, target species, number of birds tagged; see further details in Additional file 1: Table S1). Depending on the aim stated by the authors, we assigned them to six categories which reflect a growing scale ordering, from fine-scale movement decisions at flight, through medium-scale movement phases (e.g. foraging phase) and up to large-scale movement patterns such as home ranges. Specifically, these categories are: 1) flight, 2) natal dispersal phase, 3) commuting phase, 4) foraging phase, 5) migration phase, and 6) home range (Fig. 1). We also included a section for articles where the authors stated that their research focus was on management and conservation of vulture species and another for those associated with methodological developments. It is important to note that one article could contribute to more than one category and, therefore, that the sum of articles across all categories exceeds the total number of those reviewed.

## Results and discussion

### Literature and data available

**Fig. 2:**
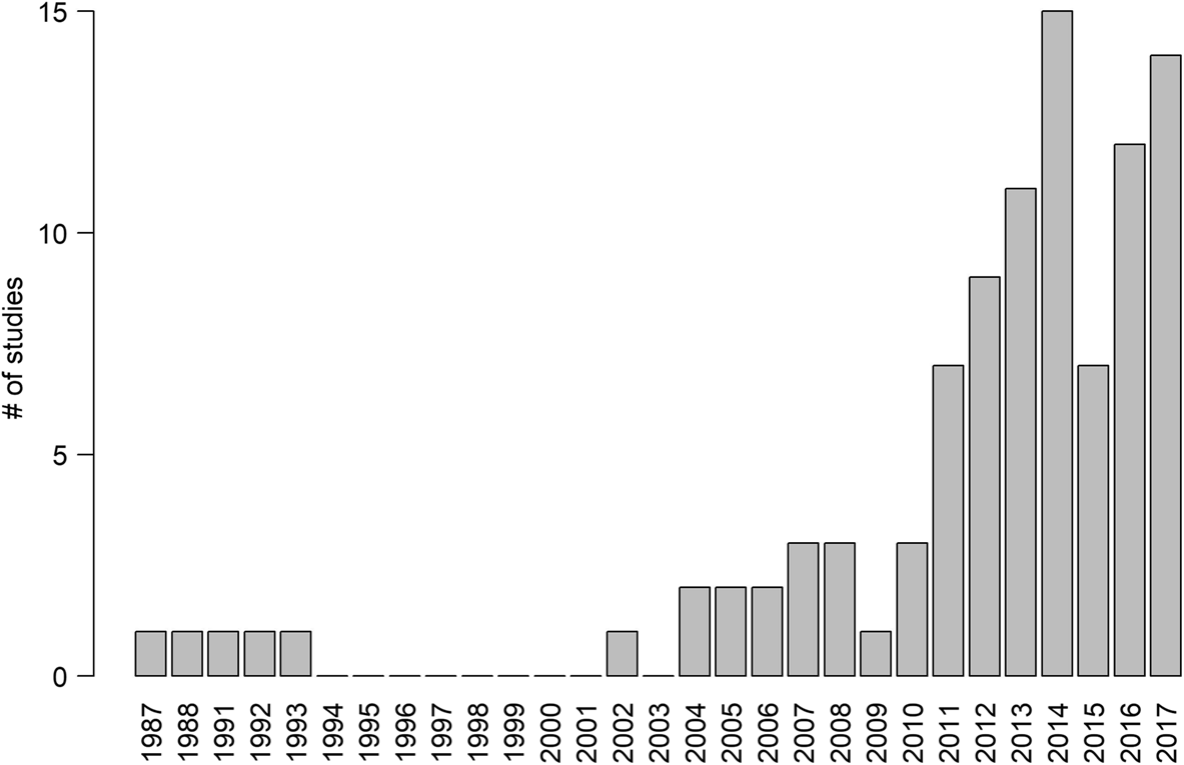
Yearly number of telemetry studies on vultures and condors published between 1987 and 2017

**Fig. 3:**
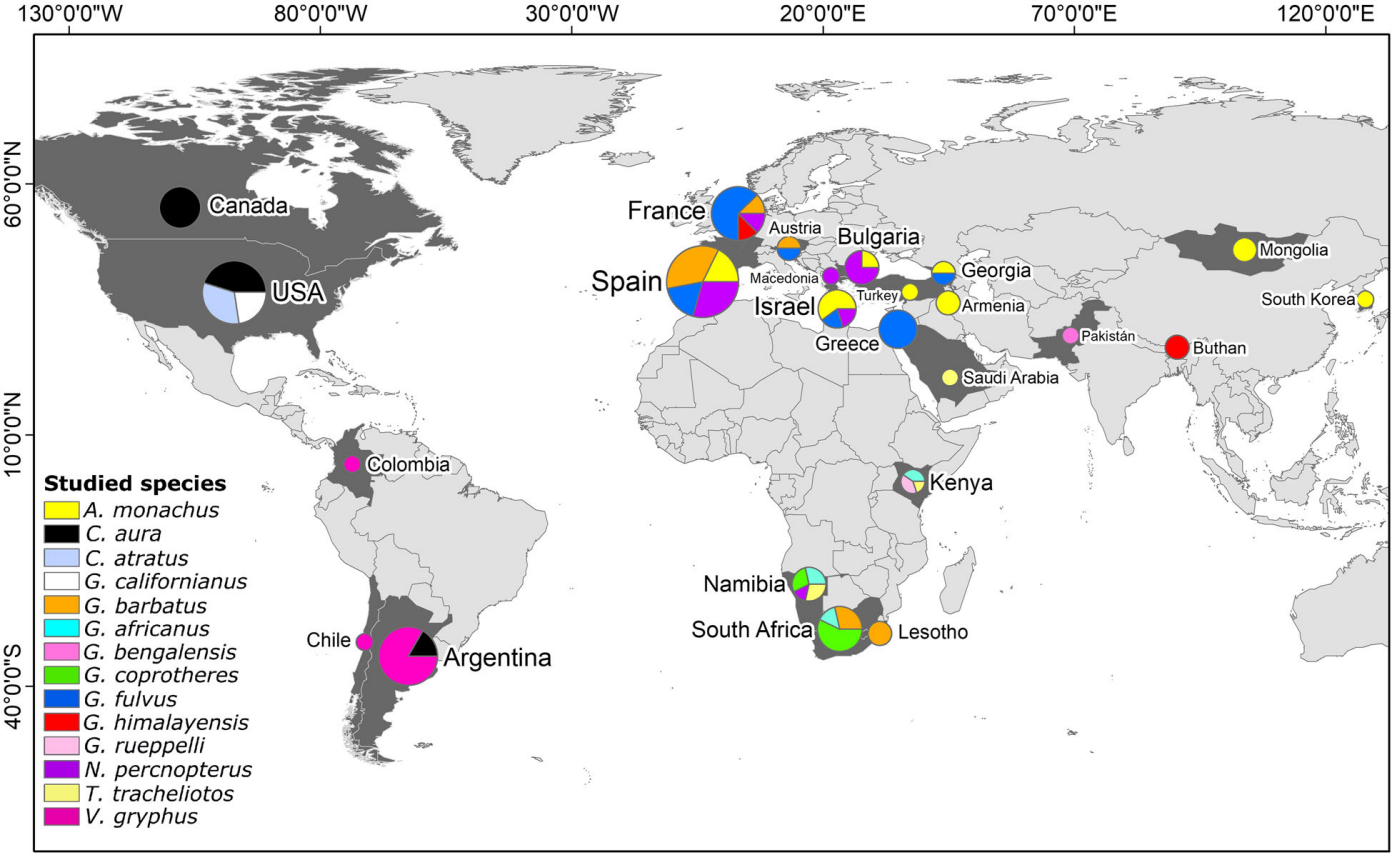
Map showing tagging locations of reviewed telemetry studies published between 1987 and 2017. The size of pie charts is proportional to the number of studies

We found a total of 97 scientific articles covering a 30-year period (1987–2017), yet with an important increase occurring during the last decade (Fig. 2). Most of these studies were published in ornithological journals followed by broad-scope and ecological journals (Additional file 2: Figure S1). As a whole, these studies reported on individuals of 14 vulture species (61% of the extant species) tagged in 24 countries, with Spain, USA, France and Argentina showing the highest contribution (Fig. 3). The Turkey Vulture (*Cathartes aura*) and the Andean Condor (*Vultur gryphus*) were the most frequently studied species among New-World vultures, whereas the Eurasian Griffon Vulture (*Gyps fulvus*) and the Bearded Vulture (*Gypaetus barbatus*) predominated among Old-World species. Of the nine species currently listed as critically endangered [19], only four of them have been studied using telemetry, with no such data for the remaining five (Additional file 3: Table S2*)*.

The reviewed studies were conducted on an average of 14 individuals, but varying from anecdotal reports on movement patterns of a single individual to studies well supported by data from up to 76 individuals (Additional file 1: Table S1). The ratio of juveniles to adults was skewed to adults (0.38:0.62; based on 1095 individuals from 83 studies), whereas the ratio of males to females was more balanced (0.43:0.47; based on 821 individuals from 50 studies). Satellite-based telemetry was the technology most frequently used to track vulture movements (93% of the reviewed studies) and VHF-based telemetry and radar technology were only occasionally implemented. In most studies (78% of the reviewed studies), transmitter devices were attached to the birds using a backpack-style harness, whilst the rest included patagial attachment and leg-loop and pelvic harnesses. The number of animal locations provided by these technologies varied from almost no data to 4.4 million data points collected over what ranged from a few days to up to seven years.

### Insights into behavior and ecology

#### Flight as the basis of movement

Flight is the primary mode of locomotion used by vultures and consequently a cross-cutting element in all movement phases discussed below. The compiled studies in this section focused on the energetics and three-dimensional analysis of vulture flight paths. In particular, five studies implemented telemetry techniques to specifically explore the flight energetics of the Eurasian and Himalayan Griffon Vultures (*Gyps himalayensis*) and of the Andean Condor, whereas other seven studies focused on three-dimensional description of flight paths of Black Vultures (*Coragyps atratus*) and Turkey Vultures (Table 1).

**Table 1:** Research effort made on each studied category considering the number of species and individuals tagged, research approaches used, mechanistic components studied and number of studies

Category	Species (# individuals)	Dominant approach (phenomenological/mechanistic)	Mechanistic components	Number of studies
Flight	*Gyps fulvus* (69) Gyps himalyensis (40) *Gymnogyps californianus* (39) *Cathartes aura* (> 19) *Coragyps atratus* (> 11) *Vultur gryphus* (10)	N/A	N/A	12
Natal dispersal phase	*Gypaetus barbatus* (> 50) *Aegypius monachus* (30)	Phenomenological	N/A	8
Commuting phase	*Gyps fulvus* (> 76) *Vultur gryphus* (23)	Mechanistic	Motion capacity Navigation capacity External factors	3
Foraging phase	*Gyps fulvus* (> 103) *Gyps coprotheres* (43) *Gyps africanus* (> 32) *Gypaetus barbatus* (29) *Aegypius monachus* (24) *Torgos tracheliotus* (> 20) *Gyps rueppelli* (12) *Gyps bengalensis* (6) *Neophron percnopterus* (< 6) *Gymnogyps califonianus* (83) *Coragyps atratus* (33) *Cathartes aura* (28) *Vultur gryphus* (23)	Phenomenological and mechanistic	Internal state Motion capacity Navigation capacity External factors	25
Migration phase	*Neophron pernocterus*(> 32) *Aegypius monachus* (19) *Gyps himalyensis* (18) *Gyps fulvus* (5) *Cathartes aura* (> 34)	Phenomenological and mechanistic	Internal state Motion capacity External factors	19
Home range	*Gyps fulvus (58)* *Gyps coprotheres* (43) *Aegypius monachus (39)* *Gypaetus barbatus (29)* *Gyps himalyensis (18)* *Neophron percnopterus (7)* *Gyps bengalensis (6)* *Torgos tracheliotus (2)* *Gymnogyps californianus (83)* *Cathartes aura (> 50)* *Coragyps atratus (> 35)* *Vultur gryphus* (23)	N/A	N/A	33
Management and conservation	*Gyps fulvus (> 79)* *Aegypius monachus (> 44)* *Gypaetus barbatus (> 41)* *Gyps coprotheres (30)* *Neophron percnopterus (27)* *Gyps africanus(6)* *Gyps bengalensis (6)* *Gyps himalyensis (18)* *Gymnogyps californianus (> 122)* *Vultur gryphus (44)* *Coragyps atratus (> 39)* *Cathartes aura (> 16)*	N/A	N/A	24
Methodological development	*Gyps fulvus (> 58)* *Gyps rueppellii (6)* *Torgos tracheliotos* (5) *Gyps africanus* (5) *Gyps himalayensis* (1) *Gymnogyps californianus (> 51)* *Coragyps atratus (19) <* *Cathartes aura* (20) *Vultur gryphus* (6)	N/A	N/A	13

Values preceded by the symbol ‘>’ represent minimum estimations. N/A means ‘not applicable’. See complete list of references in Additional file 1: Table S1

The way large-bodied vultures manage the physical and metabolic challenges of flight has inspired numerous studies in the last decades using both direct and remote tracking of birds [12, 20, 21]. Modern telemetry studies are now examining the energetic costs of flight in considerable detail taking into account the added complexity of soaring, an energy-saving mechanism that uses ascending air currents to gain lift in flight. For example, by attaching accelerometers, heart-rate loggers and GPS devices to free-flying individuals, a quantitative examination of the costs of soaring and flapping flight was conducted on one Eurasian Griffon Vulture and one Himalayan Vulture [12]. The costs of soaring were estimated in 1.43 times the basal metabolic rates (lower than theoretically expected) but increased three-fold when the birds flapped their wings to take-off or land. In this study, soaring flight mostly occurred under sunny conditions, thus adding evidence that the spatiotemporal distribution of physical energy provided by the environment is ultimately pivotal in modulating movement patterns and energy budgets of these large flying birds [22]. In fact, another telemetry study conducted on free-living Andean Condors suggested that the pattern of uplift harvesting could be comparable to the pattern of patch use during food searching; as the time spent in patchy uplifts depends on the rate of potential energy gain which decays with time [23]. Superimposed on these general strategies are state variables such as the age of birds. For example, adult Eurasian Griffon Vultures were found to show higher soaring-gliding efficiency, lower proportion of flapping flight, and lower energy expenditure during flight in comparison with juveniles birds [24]. As a whole, these studies highlight the value of considering not only the need of harvesting chemical energy from food, but also of exploiting the physical energy provided by the environment [22].

Studies focusing on the three-dimensional analysis of vulture movements pursed different purposes. Most of them were aimed at providing assessments on vultures’ collision risks with human-made structures by analyzing the range of altitudes of in-flight locations and relating it to both landscape and weather variables [25–28]. In general, results indicate that flight altitude is dependent on variables such as the time of day, uplift strength and season, but they only occasionally exceed 400 m above ground level (see also the Management and Conservation section). Other studies explored vulture flight behavior in relation to uplift availability, making special efforts to develop spatiotemporal models of uplift distribution [22, 29, 30]. These models are bringing us closer to obtaining actual energy landscapes (i.e. in the form of maps) to answer both theoretical and applied ecological questions.

Flight research is an active, promising field where studies conducted on vulture species have been pioneering in their attempt to implement new technologies to answer long-standing questions. The technologies required to monitor the biomechanical, physiological and behavioral responses of animals in flight and their energetic implications are being continuously improved [12, 31, 32]. At present, a widespread and immediate challenge to be tackled seems to be the development of user-friendly, data processing software [33] that allows practitioners to extract insights from the overwhelming amount of information provided by new technologies. Along with this, it will also be important to fuel recent efforts that are increasing our ability to characterize the environment at spatiotemporal scales that match the resolution of movement data collected to date [34]. With this, flight research will most likely bring important benefits to animal ecology and conservation, but also to engineering projects aimed at improving the operational capabilities of flying artifacts such as unmanned aerial vehicles [35, 36].

#### Movement phases

##### Natal dispersal phase

Natal dispersal involves the movement of an individual away from its birth site to another location where it will settle and reproduce [37]. Given that most vulture species experience delayed sexual maturity, natal dispersal is a long and complex process in which individuals should progressively gain flight skills and become familiar with environmental and social contexts. As a result, during this period a number of progressive ontogenetic shifts occur, modifying motivations, motion and navigation capacities and ultimately individual movement behavior. So far, nine studies conducted on the Cinereous Vulture (*Aegypius manachus*) and the Bearded Vulture focused on this movement phase (Table 1). They prioritized the description of movement patterns (phenomenological approach) over processes (mechanistic approach), as the emphasis was placed on estimating dispersal capacity by measuring a set of movement-related metrics of birds during their earliest years (0–3 yrs. old birds).

Vulture dispersive behavior predictably shows differences at both population and individual levels. For instance, Cinereous Vultures tagged in Spain spent the entire dispersal phase in the surroundings of the natal area (‘small-scale dispersants’), whereas those tagged in central Europe (Turkey, Armenia and Georgia) took long trans-continental migratory journeys to wintering grounds as soon as two or three months after fledging (‘large-scale dispersants’) [38–41]. Individual-level differences are also apparent in the movements of small-scale dispersants for both the Cinereous Vultures as well as juvenile Bearded Vultures. Individuals of both species differed in the return frequencies to birth sites, daily traveled distances, home range sizes and total dispersal distances [39, 42]. Similarly, large-scale dispersants of Cinereous Vultures differed in the migration itinerary, particularly related to departure time and migration route [41]. Finally, intra-individual differences seem to be consistent with the expected progressive ontogenetic shifts as many of the studied birds were found to take more and farther exploratory flights from their birth sites when getting older (e.g. [39]; [43]). Overall, the studies above provide realistic metrics that will serve, for instance, to inform modeling of population dynamics and scale-up consequences of vulture natal dispersal. However, further research is needed to gain in-depth knowledge of dispersal movements of this group of species.

Future studies on natal dispersal may wish to focus on two main information gaps. First, it is important to extend the study of natal dispersal to other vulture species in order to reveal the existence of species-specific dispersal strategies. Second, further studies need to be conducted over sufficiently long time periods that cover the whole dispersal process and envisage subsequent movement processes such as breeding dispersal. To date, studies have usually been short in their duration and as such have missed vital information such as the sites where the studied individuals finally settle. This information is important to connect to other processes in ecosystems (e.g. dispersal genetics, population dynamics), but also to know whether dispersants are moving into well-conserved or declining populations. The development of low-cost tracking technology (including both GPS units and attachment systems) ensuring long operating lifetime is a required condition to fulfill this gap.

##### Commuting phase

Environmental heterogeneity many times requires animals to connect geographically separated areas that provide them with essential resources such as food, water and refuge. Many species do this by adopting central place foraging [44]; i.e. they routinely commute between a particular place such as a breeding or roosting site and distant locations where resources occur. While most of vulture species behave as central-place foragers, commuting phase has been rarely seen as an individually relevant movement phase. In fact, it was the aim of only three of the reviewed studies to examine the movement decisions involved in these commuting flights and these focused on only two species; the Eurasian Griffon Vulture and the Andean Condor (Table 1).

The interaction between motion capacity and external factors has been the focus of the studies dealing with the commuting phase of vultures (Table 1). This movement phase often involves long-distance flights that birds seem to synchronize with different environmental conditions to benefit from the highest possible energy harvesting. For example, a recent study found that Andean Condors, a sexually dimorphic species, schedule their daily routines to balance the chances of exploiting food and wind resources, which was in part achieved by aligning long-distance, energy-demanding inbound flights with the times of day when the most profitable wind conditions occurred [45]. This study also showed that the sex and body size of individuals influence this trade-off solution. Similarly, another study showed that Eurasian Griffon Vultures, a species without sexual dimorphism, balance time, energy and risk constraints differently depending on whether they are taking an inbound or outbound flight [46]. During inbound flights these birds seek to minimize time and energy at the cost of flying in a more risk-prone manner when compared to outbound flights. This may be a result of the lower uncertainty concerning the destination. In this case, breeding status and age but not sex explain the inter-individual differences [46]. These findings suggest that vultures need to solve a number of dilemmas during commuting flights, which could be fertile ground for new research.

While not especially focused on the commuting phase, a recent study provided insights into mechanisms that Eurasian Griffon Vultures use to navigate and orient their outbound flights [10]. In particular, this study presented empirical evidence for the Information Centre Hypothesis (ICH, see [47]) which suggests that individuals exchange information about the location of food and use it to decide upon the route of their foraging trips. By tracking movements of dyads of foraging birds, the authors showed that individuals with no information regarding food location synchronized the departure time from roosting sites with those of informed individuals and followed them closely once in route [10]. This work suggests that external information in the form of social information plays a role in the shaping of movement paths during the commuting phase.

In summary, we suggest that the commuting phase should be considered as an individually relevant movement phase in its own right in the study of vulture movement. While this phase is often seen as a part of the foraging phase, the available evidence suggests that commuting individuals often pursue different short-term goals and balance different constraints. This is particularly true in the case of species that forage far away from their breeding or roosting sites and, therefore, show long and easily recognizable commuting flights (e.g. Andean Condors; see [45]). Telemetry data describing the commuting phase already exist for many vulture species and, therefore, achieving greater knowledge will require exploring movement data accordingly. It is worth mentioning that studies to date prioritized mechanistic approaches and largely overlooked the phenomenological description of commuting phase (Table 1). And so, to truly appreciate the complexities of commuting movements, the report of basic flight statistics should not be ignored.

##### Foraging phase

A major challenge in animal ecology is to further our understanding of how animals search for food and which variables determines search efficiency [48]. In this sense, the study of vulture searching behavior has been the centre of much theoretical and empirical work as these species depend on a single food resource (i.e. animal carcasses) which is both ephemeral and only partially predictable in space and time [49, 50]. As a consequence, a large proportion of the compiled studies focused on this phase. Overall, a total of 25 studies that collected data from more than 400 individuals of 14 species evaluated the foraging phase using both phenomenological and mechanistic approaches (Table 1).

The relevance of internal state in determining an individual’s foraging decisions is self-evident, but collecting empirical evidence from wild-ranging vultures is challenging due to the hidden and dynamic nature of animal physiology. Fortunately, telemetry studies are fulfilling this information gap. For example, researchers in Israel combined GPS location with accelerometer data from Griffon Vultures to infer feeding events and estimate food deprivation periods, a proxy of hunger [14]. They found that these birds show non-monotonic movement patterns when faced with increasing hunger levels, changing from an intensive search strategy (which maximizes food intake) to a more restrictive one (which avoids the physiological collapse due to locomotion costs). This is an example of how vultures can alternate between condition-dependent movement strategies which have direct implications on the fate of individuals. Based on these insights, and on the development of numerous bio-loggers, it is clear that further research aimed at empirically addressing the role of hunger and its cascade effects on individual behavior will improve our understanding of how foraging vultures make movement decisions.

While not exactly aimed at exploring how vultures obtain and use external information to navigate during foraging phase, a number of studies have associated their results with vulture ability to sense contextual information. For example, the species-specific sensory machinery that guides foraging individuals appears to confer different searching food efficiency to both Old- and New-World vultures. A study carried out in USA suggests that Black Vultures have comparatively inferior search abilities compared to Turkey Vultures, presumably due to lower olfactory capacity [51]. Similarly, in Namibia, Lappet-faced Vultures (*Torgos tracheliotus*) seem to be more efficient searchers than White-backed Vultures (*Gyps africanus*) due, in part, to a wider detection visual range [52]. As outlined above, the use of social information to direct food searching patterns in movement was also addressed in the context of the ICH [10]. Interestingly, a study empirically addressed questions linked to the information acquisition and use by wild foraging vultures by taking advantage of the telemetry tools that allow for the movement paths of different birds to be observed simultaneously, and in detail, as they fly in the same airspace [10]. Well-designed research to this end will help to move forward this fascinating aspect of vulture behavior.

Regarding external mechanistic components affecting foraging decisions of vultures, food-related variables are the most studied. The challenge of feeding on a partially predictable distributed resource should cause these birds to adopt foraging strategies that reduce the uncertainty as much as possible. Recent evidence shows that in environments with relatively little disturbance, vultures accomplish this by tracking the same optimal environmental conditions that increase herd mortality rates, and not by following herds [53]. This was especially the case for the White-backed, Lappet-faced and Ruppell’s Vultures (*Gyps rueppellii*) in the Mara-Serengeti ecosystem. These vultures were found to stay close to migratory herds of wildebeest only during the dry season, when herd mortality is high. By contrast, during the wet season two of these species (Ruppell’s and Lappet-faced Vultures) prioritized the use of drier over wetter areas, even when the former had lower abundance of animals. However, it is important to note that the reduction of spatiotemporal unpredictability of food is no longer a challenge for many vulture populations as, in recent years, they have become increasingly dependent on human-managed resources [54, 55]. This is especially the case for those populations that now depend on supplementary feeding stations. While foraging patterns of certain species appear to be unaltered by the availability of food at feeding stations [56–59], for other species, movements are clearly conditioned, either pushing individuals to take longer forays to feed on supplementary stations [60] or restrict food searching to those places [61]. The high abundance of alternative food sources and diet plasticity of certain populations (e.g. Bearded Vultures in the Pyrenees) could be the basis for maintaining unaltered movement patterns in places where feeding stations exist [58]. Interestingly, an environment with  high abundance of food also appears to explain the virtually unchanged movement responses of Andean Condors after an explosive volcanic eruption occurred in Patagonia [62].

##### Migration phase

Migration involves periodic and large-scale movements commonly associated with competition and seasonal changes in weather and food sources [63]. Among vultures, a total of six species are known to have migratory populations [64]. So far, 19 telemetry studies totaling at least 108 tagged individuals were conducted on the Egyptian Vulture (*Neophron percnopterus*), the Cinereous and Griffon Vultures and the Turkey Vulture (Table 1). These studies mostly described the itinerary and routes of migrating vultures and explored the role of some internal and external mechanistic components that shape the migration phase of this group of species.

Old-World vulture species were found to use different flyway corridors during their migration, routes that differed according to their tagging location. For example, Egyptian Vultures tagged in Spain and France connected to Western Africa by crossing the strait of Gibraltar and Sahara desert to finally winter close to the border between Mauritania and Senegal [65–67]. These birds covered total distances of between 2700 and 4000 km spending an average of 14 days on route. By contrast, Egyptian vultures tagged in Bulgaria, Greece, Macedonia and Albania migrated into Africa crossing Turkey and the Sinaí Peninsula to winter over a vast area of Sahel in the east of the continent [67–69]. These journeys covered distances of between 3500 and 5400 km and migration took an average of 34 days. In general, southward autumn migrations of Egyptian Vultures were similar to northward spring migrations in terms of itinerary and routes used. Other studies reported on the migration of Cinereous Vultures tagged in Georgia, Armenia and Turkey. These individuals moved from their respective countries into the Arabic Peninsula covering total distances of up to 2500 km [40, 41, 70]. Similarly, in the New World, satellite-tracked Turkey Vultures used migration routes that connect high latitudes in South- and North-America to Equatorial latitudes [9]. Mean overall distances traveled during outbound migration ranged from ~ 1300 to 5300 km and took an average of between 14 and 44 days, whereas distances registered during return migration ranged from ~ 1200 to 8300 km and took an average of between 25 and 56 days [9].

Beyond the tracking of locations, the detailed tracing of migration routes is facilitating the examination of the mechanistic bases of the vulture migration phase. The role of both physiological and morphological variables as internal mechanistic components of migration was explored using telemetry technologies. By using an inter-peritoneal data logger, for instance, researchers measured the heart rate of a migrating Turkey Vulture and estimated the physiological cost of movement [13]. They found that the heart rate of the studied bird only slightly increased with distance traveled, demonstrating the highly efficient flight performance of this long-distance migrating species. Similarly, a recent study also found that migrating Turkey Vultures showed differences in a set of movement-related metrics (i.e., distance, duration, speed and altitude of migration) as a result of differences in wing loading, which suggests that wing morphology could influence the selection of migration routes [71]. Among the internal mechanistic components that shape vulture migration, navigation capacity remains largely unexplored. However, the high mortality observed in Egyptian Vultures as they migrate from Western Europe to Africa has been linked to poor navigation capacity for some individuals [69].

The best-studied external mechanistic components to influence the migration phase were the environmental variables related to the wind. Both horizontal and vertical components of the wind have been found to shape vulture migration patterns. For example, one study found that tailwinds enhanced the rate of forward movement of migrating Egyptian Vultures during their journeys to Southern Europe and Central Africa, whereas crosswinds caused directional drifts that ultimately led birds to occupy a large area in Central Africa during winter [72]. On the other hand, studies conducted on Turkey Vultures invariably show that geographically distant populations exploit orographic and thermal uplifts according to their availability [30]. Winds are an important energetic resource for long-distance migrating vultures that, in occasions, has been suggested to determine survival rates [73]. Because of this, the use of well-informed wind scenarios is essential to move forward in the study of ecology and conservation of migrating vultures.

#### Home ranges as emerging properties of movement phases

Animals’ home ranges are commonly defined as the macroscopic spatial expression of fine-scale movement decisions [74, 75]. According to this definition, home ranges should not be considered at the same level as the movement phases discussed above, but rather as emerging properties from such phases. This conceptualization has fostered the development of a number of home range estimates which are often used as summary metrics of animal movements from both conservation and ecological perspectives [76]. So far, home-range characteristics of 12 vulture species have been reported in 33 articles (Table 1).

A large part of the research describing vultures’ home ranges were phenomenological studies aimed at contributing to vulture conservation. In general, these studies focused on little-known or threatened species and often present the first known movement datasets for the studied species. Examples include studies conducted on California Condors (*Gymnogyps californianus*) [77], Andean Condors [11, 78, 79], Bearded Vultures [57, 80], Cape Vultures [81] and Lappet-faced Vultures [82]. These works explored movement data mainly in relation to the use of protected areas [11, 57, 81, 83] and supplementary feeding stations [56, 60, 61, 84, 85].

From an ecological perspective, almost every movement phase discussed above has been individually characterized using home range analysis, with the size of the home range being the most frequently studied feature. For example, home range estimates were used to describe the progressive increase in the area explored by dispersant birds [86, 87], to delineate foraging ranges [83], and even to summarize long-distance movements such as those involved during migration [88]. Considering the values reported for the reviewed studies, home range size for vultures averages 43,991 km^2^ ranging from 5.8 to 867,811 km^2^ (based on 372 home range estimates of individual birds, see Additional file 4: Table S3). The common biological causes to explain the variability in home range size include the species identity and individual traits such as age and sex [57, 79, 81, 88, 89], but also periodic environmental fluctuations such as those related to seasonality [78, 83, 90].

The methodology used to collect and analyze data in each study, however, may influence home range estimates and the interpretation of the findings. Concurrent with the development of animal tracking technologies, alternative methods for home range estimation were developed especially to accommodate the features of available data [76, 91]. As a result, home range estimators at present vary from simple Minimum Convex Polygons to sophisticated utilization distributions based on movement models (e.g., dynamic Brownian bridge movement models). In particular, the articles we reviewed used seven different estimators: Minimum Convex Polygon at 100% (19 studies), Minimum Convex Polygon at 95% (4 studies), Fixed Kernel Density estimator 99% (4 studies), Fixed Kernel Density estimator 95% (20 studies), Fixed Kernel Density estimator 50% (1 study), Adaptive Kernel Density estimator 95% (1 study) and Brownian Bridge Movement Model estimator 95% (1 study). While the evolution in home range estimators is allowing researchers to extract more robust insights from the new movement data, the lack of consistency in the analytical methods used makes difficult to compare studies. In order to tackle this difficulty, we think that researchers should first use the most powerful and appropriate estimator for their dataset [91], but should also report estimates computed with the most frequently used methods to date (e.g. Minimum Convex Polygon 100% and Fixed Kernel Density estimator 95%). By making all estimates available to the community, future studies will be able to invest extra efforts to make these estimates comparable.

### Insights into management and conservation

To build well-founded management and conservation plans for a given species it is fundamental to know how individuals move through the space [2], and what factors influence such movements. For wide-ranging animals like vultures, satellite-based telemetry can provide such information. Thus, this tool is now contributing to assess and mitigate old and new vulture-human conflicts as well as to rethink traditional approaches to manage and conserve vulture populations [92]. Twenty-four of the reviewed studies conducted on 12 vulture species were aimed at providing information for management or conservation of these species and their environment (Table 1).

As well as informing new plans, recent telemetry studies have in some cases revealed movement patterns that question the effectiveness of traditional practices with regard to protecting areas to benefit the conservation of vulture species. The huge movements of these species easily exceed the limits of protected areas leaving birds exposed to poorly regulated decisions. This became clear in the cases of Andean Condors in Patagonia and Cape Vultures in Southern Africa. By using GPS-tracking telemetry, researchers determined that Andean Condors that breed in one country need to cross international and national borders to reach their foraging areas each day [11]. Similarly, Cape Vultures that breed inside extensive protected areas periodically forage on private rangelands [93]. The authors of these studies agreed that neither the protected areas nor private farms alone can guarantee for serious control or management practices and thus called for coordinated cross-border conservation strategies to be agreed on. Even the extensive network of protected areas established to protect vultures across the whole of Spain (covering ~ 300,997 km^2^) has been found to be insufficient [92]. However, when coordinated, the efforts of governmental institutions and of private farmers were shown to provide an improvement in the sanitary regulation of areas used by scavengers as well as important co-benefits such as the reduction of greenhouse gas emission [92].

The risk of collision for flying animals with human-made structures has notably increased during recent decades and vultures are not exempt from this new threat [94]. For this reason, the study of vulture flight aimed at preventing collisions with human infrastructures has been the focus of several investigations. For example, efforts have been made to inform wind turbine placements by considering both the landscape features associated with different patterns of habitat use [28, 95] and the three-dimensional models of vulture flight that estimate collision risk and infer mortality [27, 96, 97]. Worryingly, most of the studies show high spatial overlap between wind turbine projects and the areas used by vultures; sometimes involving vulnerable populations such as those of California Condors in USA [28], Bearded Vultures in southern Africa [95] and Cinereous vultures in south-eastern Europe [96]. Similar efforts have also been made to prevent vulture-aircraft collisions, particularly in the cases of Turkey and Black Vultures in USA [25, 26, 98]. Finally, the complex association between vultures and power lines was addressed in the case of Cape Vultures from South Africa [93]. In this case, there is evidence that the movement patterns of these birds are closely linked to the spatial extent of transmission power lines and, according to the authors of such study, these human structures seem to provide ideal roosting sites for these birds, but are also responsible for an increased mortality.

### Methodological developments

Several telemetry studies on vultures have been conducted with the aim of testing and improving different aspects of telemetry devices. For instance, some studies were focused on the development of harness attachment techniques that prevent transmitter loss and at the same time reduce stress to birds [99]. Similarly, others evaluated the effects of environmental conditions and animal behavior on performance of solar-powered GPS-GSM transmitters [100] or compared the benefits of satellite telemetry versus avian radar [101]. The usefulness of GPS-GSM transmitters to evaluate mortality was also addressed [102], making special effort to determine mortality causes. Finally, data from tagged vultures were also used to test and calibrate new analytical tools such as the identification of behaviors from accelerometer data [33, 103–105].

## Concluding remarks

Telemetry studies have revealed amazing details of vulture movements paving the way for new research in vultures’ biology. As a result, we now have highly accurate measurements of flight energetics and a better understanding of the morphological, physiological and context-dependent drivers that underlie the movement decisions of these birds. While there is still much to be learned, the information available can be used to scale-up the consequences of movement, such as population dynamics and disease ecology. Likewise, telemetry-based movement research has allowed elaborating preventive analyses to adequately protect these species and their environments (e.g. from wind farms) as well as rethinking conservation strategies to improve their efficiency. This new knowledge should be considered in the decision making of natural resource management.

We need to widen the net and expand the species considered. To date, 40% of vulture species have not been tracked using telemetry techniques, which may be concealing ecological processes as well as conservation issues in which these species are involved. This is particularly relevant for threatened species, as this technology could reveal key information regarding their survival. Our review shows that some vultures’ movement phases (e.g. foraging phase) have been well covered by scientific literature, but that other equally prominent movement phases have been relatively poorly studied (e.g. dispersal phase). Moreover, we see a need for more mechanistic approaches particularly as we go on to consider the role of internal variables (e.g. navigation skills) in shaping movement decisions of individual vultures. For this, it will be crucial to promote the extensive use of already existing data (e.g. in Movebank: https://www.movebank.org/) as well as the development of collaborative projects among scientists from different disciplines and technology developers. We envision an exciting research field in which the synergy between telemetry technologies and multidisciplinary and comprehensive approaches will unveil valuable but still little-understood aspects of vulture biology and their environments.

## Additional files

Additional file 1: **Table S1.** Scientific literature about telemetry studies published on vultures between 1987 and 2017. (XLSX 23 kb)

Additional file 2: **Figure S1.** Scientific journals on which vulture tracking studies were published between 1987 and 2017. (DOCX 256 kb)

Additional file 3: **Table S2.** Conservation status of the 23 extant vulture and condor species according to the Red List of Threatened Species (IUCN 2017). Species not registering telemetry studies are marked with an asterisk. (DOCX 17 kb)

Additional file 4: **Table S3.** Home-range estimates reported for telemetry studies conducted on vultures and condors between 1987 and 2017. (DOCX 65 kb)
