# Supplementary figures and images for: A three-decade review of telemetry studies on vultures and condors
Source: Mov Ecol. 2018 Sep 4;6:13. doi: 10.1186/s40462-018-0133-5 (PMC6122777; doi:10.1186/s40462-018-0133-5)

**Figure S1.** Scientific journals on which vulture tracking studies were published between 1987 and 2017.


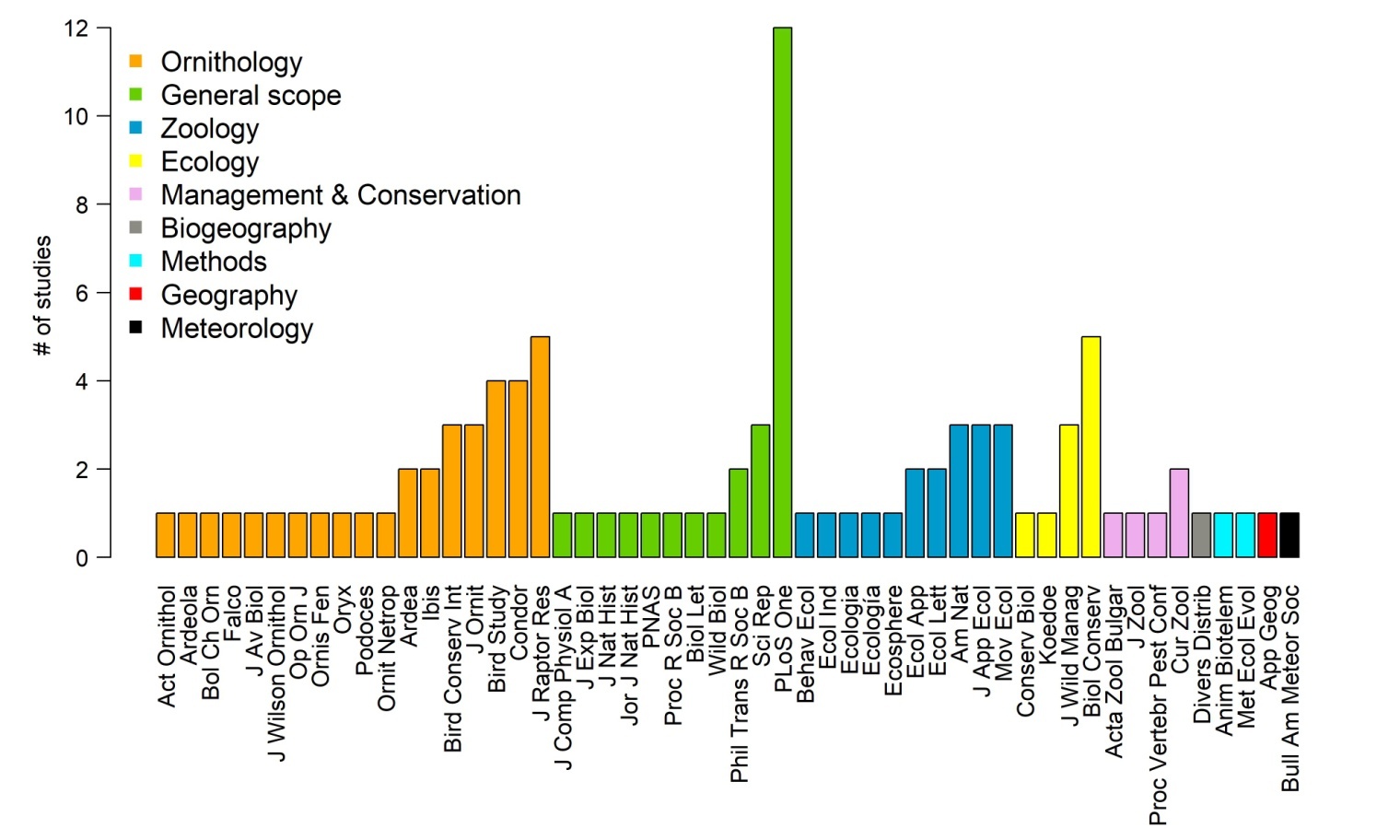

Supplement: Supplementary file 2 — Figure S1. Scientific journals on which vulture tracking studies were published between 1987 and 2017. (DOCX 256 kb) [file 40462_2018_133_MOESM2_ESM.docx]
