# Supplementary material for: A three-decade review of telemetry studies on vultures and condors
Source: Mov Ecol. 2018 Sep 4;6:13. doi: 10.1186/s40462-018-0133-5 (PMC6122777; doi:10.1186/s40462-018-0133-5)
Supplement: Supplementary file 3 — Table S2. Conservation status of the 23 extant vulture and condor species according to the Red List of Threatened Species (IUCN 2017). Species not registering telemetry studies are marked with an asterisk. (DOCX 17 kb) [file 40462_2018_133_MOESM3_ESM.docx]

**Table S2.** Conservation status of the 23 extant vulture and condor species according to the Red List of Threatened Species (IUCN 2017). Species not registering telemetry studies are marked with an asterisk.

|  |  |  |
| --- | --- | --- |
| **Popular name** | **Scientific name** | **Conservation status** |
| Griffon vulture | *Gyps fulvus* | Least Concern |
| Palm-Nut vulture | *Gypohierax angolensis* | Least Concern(*) |
| Greater Yellow-Headed vulture | *Cathartes melambrotus* | Least Concern(*) |
| Lesser Yellow-Headed vulture | *Cathartes burrovianus* | Least Concern(*) |
| King vulture | *Sarcoramphus papa* | Least Concern(*) |
| Turkey vulture | *Cathartes aura* | Least Concern |
| Black vulture | *Coragyps atratus* | Least Concern |
| Cinereous vulture | *Aegypius monachus* | Near Threatened |
| Himalayan vulture | *Gyps himalayensis* | Near Threatened |
| Bearded vulture | *Gypaetus barbatus* | Near Threatened |
| Andean condor | *Vultur gryphus* | Near Threatened |
| Cape vulture | *Gyps coprotheres* | Endangered |
| Egyptian vulture | *Neophron percnopterus* | Endangered |
| Lappet-Faced vulture | *Torgos tracheliotus* | Endangered |
| Rüppell's vulture | *Gyps rueppelli* | Critically Endangered |
| White-Backed vulture | *Gyps africanus* | Critically Endangered |
| White-Rumped vulture | *Gyps bengalensis* | Critically Endangered |
| White-Headed vulture | *Trigonoceps occipitalis* | Critically Endangered(*) |
| Hooded vulture | *Necrosyrtes monachus* | Critically Endangered(*) |
| Indian vulture | *Gyps indicus* | Critically Endangered(*) |
| Slender-Billed Vulture | *Gyps tenuirostris* | Critically Endangered(*) |
| Red-Headed vulture | *Sarcogyps calvus* | Critically Endangered(*) |
| California condor | *Gymnogyps californianus* | Critically Endangered |
|  |  |  |
