# Supplementary material for: A three-decade review of telemetry studies on vultures and condors
Source: Mov Ecol. 2018 Sep 4;6:13. doi: 10.1186/s40462-018-0133-5 (PMC6122777; doi:10.1186/s40462-018-0133-5)
Supplement: Supplementary file 4 — Table S3. Home-range estimates reported for telemetry studies conducted on vultures and condors between 1987 and 2017. (DOCX 65 kb) [file 40462_2018_133_MOESM4_ESM.docx]

**Table S3.** Home-range estimates reported for telemetry studies conducted on vultures and condors between 1987 and 2017.

|  |  |  |  |  |  |  |
| --- | --- | --- | --- | --- | --- | --- |
| **Species** | **Sex** | **Age** | **Estimator** | **Size (km2)** | **Mean size (km2)** | **Reference** |
| Aegypius monachus | male | adult | kde95 | 1267.061 | 1177.0 | Carrete&Donazar_2005 |
| Aegypius monachus | male | adult | kde95 | 2886.709 |  |  |
| Aegypius monachus | male | adult | kde95 | 1624.422 |  |  |
| Aegypius monachus | male | adult | kde95 | 1275.125 |  |  |
| Aegypius monachus | male | adult | kde95 | 2388.161 |  |  |
| Aegypius monachus | male | adult | kde95 | 1096.994 |  |  |
| Aegypius monachus | male | adult | kde95 | 1302.697 |  |  |
| Aegypius monachus | male | adult | kde95 | 761.454 |  |  |
| Aegypius monachus | male | adult | kde95 | 837.3618 |  |  |
| Aegypius monachus | male | adult | kde95 | 1245.063 |  |  |
| Aegypius monachus | female | adult | kde95 | 1162.604 |  |  |
| Aegypius monachus | female | adult | kde95 | 577.0069 |  |  |
| Aegypius monachus | female | adult | kde95 | 1203.573 |  |  |
| Aegypius monachus | female | adult | kde95 | 1245.063 |  |  |
| Aegypius monachus | male | adult | kde95 | 254.821 |  |  |
| Aegypius monachus | female | adult | kde95 | 897.7144 |  |  |
| Aegypius monachus | male | adult | kde95 | 1044.136 |  |  |
| Aegypius monachus | male | adult | kde95 | 382.1506 |  |  |
| Aegypius monachus | male | adult | kde95 | 1242.597 |  |  |
| Aegypius monachus | male | adult | kde95 | 845.0705 |  |  |
| Aegypius monachus | male | NA | mcp100 | 33 | 2820.2 | Castaño etal_2015 |
| Aegypius monachus | NA | NA | mcp100 | 33 |  |  |
| Aegypius monachus | male | immature | mcp100 | 540 |  |  |
| Aegypius monachus | NA | immature | mcp100 | 744 |  |  |
| Aegypius monachus | male | immature | mcp100 | 1106 |  |  |
| Aegypius monachus | male | immature | mcp100 | 2265 |  |  |
| Aegypius monachus | female | immature | mcp100 | 2828 |  |  |
| Aegypius monachus | male | immature | mcp100 | 3209 |  |  |
| Aegypius monachus | male | NA | mcp100 | 3230 |  |  |
| Aegypius monachus | female | immature | mcp100 | 3298 |  |  |
| Aegypius monachus | male | immature | mcp100 | 6736 |  |  |
| Aegypius monachus | male | NA | mcp100 | 9820 |  |  |
| Aegypius monachus | male | NA | kde95 | 6354 | 92914.2 |  |
| Aegypius monachus | NA | NA | kde95 | 538 |  |  |
| Aegypius monachus | male | immature | kde95 | 23777 |  |  |
| Aegypius monachus | NA | immature | kde95 | 2287 |  |  |
| Aegypius monachus | male | immature | kde95 | 36395 |  |  |
| Aegypius monachus | male | immature | kde95 | 102427 |  |  |
| Aegypius monachus | female | immature | kde95 | 178850 |  |  |
| Aegypius monachus | male | immature | kde95 | 165936 |  |  |
| Aegypius monachus | male | NA | kde95 | 98206 |  |  |
| Aegypius monachus | female | immature | kde95 | 71897 |  |  |
| Aegypius monachus | male | immature | kde95 | 261104 |  |  |
| Aegypius monachus | male | NA | kde95 | 167199 |  |  |
| Aegypius monachus | female | NA | mcp100 | 269.15 | 1095.7 | Vasilakis etal_2008 |
| Aegypius monachus | male | NA | mcp100 | 369.96 |  |  |
| Aegypius monachus | female | NA | mcp100 | 414.5 |  |  |
| Aegypius monachus | male | NA | mcp100 | 576.59 |  |  |
| Aegypius monachus | male | NA | mcp100 | 666.58 |  |  |
| Aegypius monachus | female | adult | mcp100 | 809 |  |  |
| Aegypius monachus | female | NA | mcp100 | 811.6 |  |  |
| Aegypius monachus | male | adult | mcp100 | 1134.7 |  |  |
| Aegypius monachus | female | adult | mcp100 | 1241.7 |  |  |
| Aegypius monachus | male | NA | mcp100 | 1264.99 |  |  |
| Aegypius monachus | female | NA | mcp100 | 1370.38 |  |  |
| Aegypius monachus | female | adult | mcp100 | 1370.8 |  |  |
| Aegypius monachus | female | adult | mcp100 | 1742 |  |  |
| Aegypius monachus | male | adult | mcp100 | 1908.6 |  |  |
| Aegypius monachus | male | NA | mcp100 | 2485.42 |  |  |
| Aegypius monachus | female | NA | mcp95 | 197.0 | 1088.6 |  |
| Aegypius monachus | male | NA | mcp95 | 139.8 |  |  |
| Aegypius monachus | female | NA | mcp95 | 271.5 |  |  |
| Aegypius monachus | male | NA | mcp95 | 319.5 |  |  |
| Aegypius monachus | male | NA | mcp95 | 393.5 |  |  |
| Aegypius monachus | female | adult | mcp95 | 5323 |  |  |
| Aegypius monachus | female | NA | mcp95 | 626.1 |  |  |
| Aegypius monachus | male | adult | mcp95 | 812.8 |  |  |
| Aegypius monachus | female | adult | mcp95 | 827.8 |  |  |
| Aegypius monachus | male | NA | mcp95 | 1071.3 |  |  |
| Aegypius monachus | female | NA | mcp95 | 1203.1 |  |  |
| Aegypius monachus | female | adult | mcp95 | 656.4 |  |  |
| Aegypius monachus | female | adult | mcp95 | 997.5 |  |  |
| Aegypius monachus | male | adult | mcp95 | 1654.1 |  |  |
| Aegypius monachus | male | NA | mcp95 | 1835.8 |  |  |
| Aegypius monachus | female | NA | kde95 | 252.68 | 607.7 |  |
| Aegypius monachus | male | NA | kde95 | 223.7 |  |  |
| Aegypius monachus | female | NA | kde95 | 648.6 |  |  |
| Aegypius monachus | male | NA | kde95 | 235.91 |  |  |
| Aegypius monachus | male | NA | kde95 | 728.36 |  |  |
| Aegypius monachus | female | adult | kde95 | 339.6 |  |  |
| Aegypius monachus | female | NA | kde95 | 441.81 |  |  |
| Aegypius monachus | male | adult | kde95 | 478 |  |  |
| Aegypius monachus | female | adult | kde95 | 651.4 |  |  |
| Aegypius monachus | male | NA | kde95 | 1110.59 |  |  |
| Aegypius monachus | female | NA | kde95 | 599.44 |  |  |
| Aegypius monachus | female | adult | kde95 | 491.3 |  |  |
| Aegypius monachus | female | adult | kde95 | 748 |  |  |
| Aegypius monachus | male | adult | kde95 | 963.9 |  |  |
| Aegypius monachus | male | NA | kde95 | 1202.37 |  |  |
| Aegypius monachus | male | NA | kde95 | 2662.45 | 1762.7 | Vasilakis etal_2017 |
| Aegypius monachus | female | NA | kde95 | 2281.7 |  |  |
| Aegypius monachus | female | NA | kde95 | 737.5 |  |  |
| Aegypius monachus | female | NA | kde95 | 491.26 |  |  |
| Aegypius monachus | male | NA | kde95 | 1491.36 |  |  |
| Aegypius monachus | female | NA | kde95 | 2520.82 |  |  |
| Aegypius monachus | male | NA | kde95 | 1947.97 |  |  |
| Aegypius monachus | male | NA | kde95 | 1196.64 |  |  |
| Aegypius monachus | female | NA | kde95 | 1646.32 |  |  |
| Aegypius monachus | male | NA | kde95 | 2170.03 |  |  |
| Aegypius monachus | female | NA | kde95 | 1925.99 |  |  |
| Aegypius monachus | NA | NA | kde95 | 857.46 |  |  |
| Aegypius monachus | NA | NA | kde95 | 1060.23 |  |  |
| Aegypius monachus | NA | NA | kde95 | 2524.7 |  |  |
| Aegypius monachus | NA | NA | kde95 | 3917.89 |  |  |
| Aegypius monachus | NA | NA | kde95 | 2051.11 |  |  |
| Aegypius monachus | NA | NA | kde95 | 1621.31 |  |  |
| Aegypius monachus | NA | NA | kde95 | 730.04 |  |  |
| Aegypius monachus | NA | NA | kde95 | 1656.8 |  |  |
| Aegypius monachus | NA | immature | kde95 | 315.5 | 356.5 | Yamac_2012 |
| Aegypius monachus | NA | immature | kde95 | 147.7 |  |  |
| Aegypius monachus | NA | immature | kde95 | 606.4 |  |  |
| Gyps fulvus | NA | adult | mcp100 | 1450 | 13820.4 | García-Ripollés etal_2011 |
| Gyps fulvus | NA | adult | mcp100 | 2127 |  |  |
| Gyps fulvus | NA | adult | mcp100 | 3509 |  |  |
| Gyps fulvus | NA | immature | mcp100 | 4655 |  |  |
| Gyps fulvus | NA | adult | mcp100 | 10183 |  |  |
| Gyps fulvus | NA | adult | mcp100 | 10240 |  |  |
| Gyps fulvus | NA | adult | mcp100 | 21142 |  |  |
| Gyps fulvus | NA | adult | mcp100 | 57257 |  |  |
| Gyps fulvus | NA | adult | kde95 | 717 | 6557.5 |  |
| Gyps fulvus | NA | adult | kde95 | 497 |  |  |
| Gyps fulvus | NA | adult | kde95 | 4796 |  |  |
| Gyps fulvus | NA | immature | kde95 | 508 |  |  |
| Gyps fulvus | NA | adult | kde95 | 7396 |  |  |
| Gyps fulvus | NA | adult | kde95 | 3359 |  |  |
| Gyps fulvus | NA | adult | kde95 | 8755 |  |  |
| Gyps fulvus | NA | adult | kde95 | 26432 |  |  |
| Gyps fulvus | female | adult | kde95 | 1735 | 994.5 | Monsarrat etal_2013 |
| Gyps fulvus | male | adult | kde95 | 254 |  |  |
| Gyps fulvus | NA | immature | mcp100 | 206 | 471.7 | Xirouchakis etal_2009 |
| Gyps fulvus | NA | immature | mcp100 | 291.5 |  |  |
| Gyps fulvus | NA | immature | mcp100 | 538.8 |  |  |
| Gyps fulvus | NA | immature | mcp100 | 850.6 |  |  |
| Gyps fulvus | NA | immature | akd95 | 194.8 | 379.9 |  |
| Gyps fulvus | NA | immature | akd95 | 319.7 |  |  |
| Gyps fulvus | NA | immature | akd95 | 527.1 |  |  |
| Gyps fulvus | NA | immature | akd95 | 478.2 |  |  |
| Gyps coprotheres | NA | immature | kde95 | 370444 | 161419.7 | Kane etal_2016 |
| Gyps coprotheres | NA | immature | kde95 | 393788 |  |  |
| Gyps coprotheres | NA | immature | kde95 | 427971 |  |  |
| Gyps coprotheres | NA | immature | kde95 | 637 |  |  |
| Gyps coprotheres | NA | immature | kde95 | 165599 |  |  |
| Gyps coprotheres | NA | immature | kde95 | 5047 |  |  |
| Gyps coprotheres | NA | immature | kde95 | 178277 |  |  |
| Gyps coprotheres | NA | immature | kde95 | 99837 |  |  |
| Gyps coprotheres | NA | immature | kde95 | 480431 |  |  |
| Gyps coprotheres | NA | immature | kde95 | 210740 |  |  |
| Gyps coprotheres | NA | immature | kde95 | 121100 |  |  |
| Gyps coprotheres | NA | immature | kde95 | 251735 |  |  |
| Gyps coprotheres | NA | adult | kde95 | 79158 |  |  |
| Gyps coprotheres | NA | adult | kde95 | 166640 |  |  |
| Gyps coprotheres | NA | adult | kde95 | 57728 |  |  |
| Gyps coprotheres | NA | adult | kde95 | 282202 |  |  |
| Gyps coprotheres | NA | adult | kde95 | 449835 |  |  |
| Gyps coprotheres | NA | adult | kde95 | 160312 |  |  |
| Gyps coprotheres | NA | adult | kde95 | 790 |  |  |
| Gyps coprotheres | NA | adult | kde95 | 1986 |  |  |
| Gyps coprotheres | NA | adult | kde95 | 2820 |  |  |
| Gyps coprotheres | NA | adult | kde95 | 2637 |  |  |
| Gyps coprotheres | NA | adult | kde95 | 2465 |  |  |
| Gyps coprotheres | NA | adult | kde95 | 232504 |  |  |
| Gyps coprotheres | NA | adult | kde95 | 7774 |  |  |
| Gyps coprotheres | NA | adult | kde95 | 102625 |  |  |
| Gyps coprotheres | NA | adult | kde95 | 103251 |  |  |
| Gyps coprotheres | NA | adult | mcp100 | 36401 | 286386.3 | Phipps etal_2013b |
| Gyps coprotheres | NA | adult | mcp100 | 56152 |  |  |
| Gyps coprotheres | NA | adult | mcp100 | 92092 |  |  |
| Gyps coprotheres | NA | adult | mcp100 | 165337 |  |  |
| Gyps coprotheres | NA | adult | mcp100 | 258294 |  |  |
| Gyps coprotheres | NA | immature | mcp100 | 273946 |  |  |
| Gyps coprotheres | NA | immature | mcp100 | 392856 |  |  |
| Gyps coprotheres | NA | immature | mcp100 | 434588 |  |  |
| Gyps coprotheres | NA | immature | mcp100 | 867811 |  |  |
| Gyps coprotheres | NA | adult | kde99 | 53589.0 | 223132.3 |  |
| Gyps coprotheres | NA | adult | kde99 | 52385.0 |  |  |
| Gyps coprotheres | NA | adult | kde99 | 78847.0 |  |  |
| Gyps coprotheres | NA | adult | kde99 | 69254.0 |  |  |
| Gyps coprotheres | NA | adult | kde99 | 149687.0 |  |  |
| Gyps coprotheres | NA | immature | kde99 | 161311.0 |  |  |
| Gyps coprotheres | NA | immature | kde99 | 312715.0 |  |  |
| Gyps coprotheres | NA | immature | kde99 | 392719.0 |  |  |
| Gyps coprotheres | NA | immature | kde99 | 737684.0 |  |  |
| Gypaetus barbatus | male | immature | mcp100 | 945 | 8380.1 | Gil etal_2014 |
| Gypaetus barbatus | female | immature | mcp100 | 978 |  |  |
| Gypaetus barbatus | female | immature | mcp100 | 1155 |  |  |
| Gypaetus barbatus | female | immature | mcp100 | 4544 |  |  |
| Gypaetus barbatus | female | immature | mcp100 | 4929 |  |  |
| Gypaetus barbatus | male | immature | mcp100 | 7178 |  |  |
| Gypaetus barbatus | female | immature | mcp100 | 16993 |  |  |
| Gypaetus barbatus | female | immature | mcp100 | 19008 |  |  |
| Gypaetus barbatus | male | adult | mcp100 | 19691 |  |  |
| Gypaetus barbatus | male | immature | kde95 | 1796 | 8669.6 |  |
| Gypaetus barbatus | female | immature | kde95 | 1973 |  |  |
| Gypaetus barbatus | female | immature | kde95 | 3665 |  |  |
| Gypaetus barbatus | female | immature | kde95 | 6128 |  |  |
| Gypaetus barbatus | female | immature | kde95 | 6209 |  |  |
| Gypaetus barbatus | male | immature | kde95 | 5603 |  |  |
| Gypaetus barbatus | female | immature | kde95 | 8717 |  |  |
| Gypaetus barbatus | female | immature | kde95 | 21019 |  |  |
| Gypaetus barbatus | male | adult | kde95 | 22916 |  |  |
| Gypaetus barbatus | female | immature | mcp100 | 10813 | 12511.0 | Krüger etal_2017 |
| Gypaetus barbatus | female | immature | mcp100 | 14209 |  |  |
| Gypaetus barbatus | female | immature | kde95 | 11644 | 11466.3 |  |
| Gypaetus barbatus | female | immature | kde95 | 15915 |  |  |
| Gypaetus barbatus | female | immature | kde95 | 6840 |  |  |
| Gypaetus barbatus | female | immature | mcp100 | 38500 | 38500.0 | Urios etal_2015 |
| Gypaetus barbatus | female | adult | kde95 | 206 | 206.0 | Gavashelishvili etal_2007 |
| Neophron percnopterus | NA | NA | mcp100 | 16.395 | 16.9 | Pfeiffer etal_2015 |
| Neophron percnopterus | NA | NA | mcp100 | 16.396 |  |  |
| Neophron percnopterus | NA | NA | mcp100 | 16.808 |  |  |
| Neophron percnopterus | NA | NA | mcp100 | 17.947 |  |  |
| Neophron percnopterus | NA | NA | kde99 | 20.2 | 25.1 |  |
| Neophron percnopterus | NA | NA | kde99 | 27.9 |  |  |
| Neophron percnopterus | NA | NA | kde99 | 24.9 |  |  |
| Neophron percnopterus | NA | NA | kde99 | 27.3 |  |  |
| Neophron percnopterus | NA | adult | kde95 | 9596 | 20742.3 | García-Ripollés etal_2010 |
| Neophron percnopterus | NA | adult | kde95 | 26016 |  |  |
| Neophron percnopterus | NA | adult | kde95 | 26615 |  |  |
| Gyps africanus | NA | immature | mcp100 | 124492 | 297506.2 | Phipps etal_2013a |
| Gyps africanus | NA | immature | mcp100 | 144568 |  |  |
| Gyps africanus | NA | immature | mcp100 | 155301 |  |  |
| Gyps africanus | NA | immature | mcp100 | 332451 |  |  |
| Gyps africanus | NA | immature | mcp100 | 439520 |  |  |
| Gyps africanus | NA | immature | mcp100 | 588705 |  |  |
| Gyps africanus | NA | immature | kde95 | 47132 | 334923.0 |  |
| Gyps africanus | NA | immature | kde95 | 125861 |  |  |
| Gyps africanus | NA | immature | kde95 | 145854 |  |  |
| Gyps africanus | NA | immature | kde95 | 342413 |  |  |
| Gyps africanus | NA | immature | kde95 | 765483 |  |  |
| Gyps africanus | NA | immature | kde95 | 582795 |  |  |
| Gyps bengalensis | male | adult | mcp100 | 1825 | 24154.6 | Gilbert etal_2007 |
| Gyps bengalensis | male | adult | mcp100 | 4625 |  |  |
| Gyps bengalensis | male | adult | mcp100 | 5069 |  |  |
| Gyps bengalensis | male | adult | mcp100 | 40324 |  |  |
| Gyps bengalensis | male | adult | mcp100 | 68930 |  |  |
| Torgos tracheliotos | NA | immature | mcp100 | 283380 | 283470.0 | Shobrak etal_2015 |
| Torgos tracheliotos | NA | immature | mcp100 | 283560 |  |  |
| Catarthes aura | NA | adult | mcp95 | 93.0 | 547.6 | Hedlig etal_2013 |
| Catarthes aura | female | adult | mcp95 | 243.0 |  |  |
| Catarthes aura | female | adult | mcp95 | 1286.0 |  |  |
| Catarthes aura | female | adult | mcp95 | 1071.0 |  |  |
| Catarthes aura | female | adult | mcp95 | 469.0 |  |  |
| Catarthes aura | male | adult | mcp95 | 698.0 |  |  |
| Catarthes aura | male | adult | mcp95 | 731.0 |  |  |
| Catarthes aura | female | adult | mcp95 | 314.0 |  |  |
| Catarthes aura | female | adult | mcp95 | 23.0 |  |  |
| Catarthes aura | NA | adult | kde95 | 167 | 16814.4 |  |
| Catarthes aura | female | adult | kde95 | 14243 |  |  |
| Catarthes aura | female | adult | kde95 | 1895 |  |  |
| Catarthes aura | female | adult | kde95 | 1915 |  |  |
| Catarthes aura | female | adult | kde95 | 1046 |  |  |
| Catarthes aura | male | adult | kde95 | 54698 |  |  |
| Catarthes aura | male | adult | kde95 | 76731 |  |  |
| Catarthes aura | female | adult | kde95 | 581 |  |  |
| Catarthes aura | female | adult | kde95 | 54 |  |  |
| Catarthes aura | NA | adult | mcp95 | 953.0 | 371.0 | Houston etal_2011 |
| Catarthes aura | NA | adult | mcp95 | 502.0 |  |  |
| Catarthes aura | NA | adult | mcp95 | 261.0 |  |  |
| Catarthes aura | female | adult | mcp95 | 416.0 |  |  |
| Catarthes aura | male | adult | mcp95 | 47.0 |  |  |
| Catarthes aura | female | adult | mcp95 | 47.0 |  |  |
| Catarthes aura | NA | adult | kde95 | 1992 | 648.3 |  |
| Catarthes aura | NA | adult | kde95 | 891 |  |  |
| Catarthes aura | NA | adult | kde95 | 316 |  |  |
| Catarthes aura | female | adult | kde95 | 567 |  |  |
| Catarthes aura | male | adult | kde95 | 49 |  |  |
| Catarthes aura | female | adult | kde95 | 75 |  |  |
| Catarthes aura | NA | NA | mcp95 | 247.6 | 2426.5 | Dodge etal_2014 |
| Catarthes aura | NA | NA | mcp95 | 273.6 |  |  |
| Catarthes aura | NA | NA | mcp95 | 351.0 |  |  |
| Catarthes aura | NA | NA | mcp95 | 5.8 |  |  |
| Catarthes aura | NA | NA | mcp95 | 88.3 |  |  |
| Catarthes aura | NA | NA | mcp95 | 11.6 |  |  |
| Catarthes aura | NA | NA | mcp95 | 11.7 |  |  |
| Catarthes aura | NA | NA | mcp95 | 12.0 |  |  |
| Catarthes aura | NA | NA | mcp95 | 130.5 |  |  |
| Catarthes aura | NA | NA | mcp95 | 105.0 |  |  |
| Catarthes aura | NA | NA | mcp95 | 37.9 |  |  |
| Catarthes aura | NA | NA | mcp95 | 38.0 |  |  |
| Catarthes aura | NA | NA | mcp95 | 53.5 |  |  |
| Catarthes aura | NA | NA | mcp95 | 12.5 |  |  |
| Catarthes aura | NA | NA | mcp95 | 110.5 |  |  |
| Catarthes aura | NA | NA | mcp95 | 110.6 |  |  |
| Catarthes aura | NA | NA | mcp95 | 12.7 |  |  |
| Catarthes aura | NA | NA | mcp95 | 38.5 |  |  |
| Catarthes aura | NA | NA | mcp95 | 100.4 |  |  |
| Catarthes aura | NA | NA | mcp95 | 38.6 |  |  |
| Catarthes aura | NA | NA | mcp95 | 12.9 |  |  |
| Catarthes aura | NA | NA | mcp95 | 13.0 |  |  |
| Catarthes aura | NA | NA | mcp95 | 13.1 |  |  |
| Catarthes aura | NA | NA | mcp95 | 13.2 |  |  |
| Catarthes aura | NA | NA | mcp95 | 13.3 |  |  |
| Catarthes aura | NA | NA | mcp95 | 183.4 |  |  |
| Catarthes aura | NA | NA | mcp95 | 13.9 |  |  |
| Catarthes aura | NA | NA | mcp95 | 9.9 |  |  |
| Catarthes aura | NA | NA | mcp95 | 239.4 |  |  |
| Catarthes aura | NA | NA | mcp95 | 280.2 |  |  |
| Catarthes aura | NA | NA | mcp95 | 486.6 |  |  |
| Catarthes aura | NA | NA | mcp95 | 481.7 |  |  |
| Catarthes aura | NA | NA | mcp95 | 621.0 |  |  |
| Catarthes aura | NA | NA | mcp95 | 569.7 |  |  |
| Catarthes aura | NA | NA | mcp95 | 770.1 |  |  |
| Catarthes aura | NA | NA | mcp95 | 826.9 |  |  |
| Catarthes aura | NA | NA | mcp95 | 832.3 |  |  |
| Catarthes aura | NA | NA | mcp95 | 671.0 |  |  |
| Catarthes aura | NA | NA | mcp95 | 1192.8 |  |  |
| Catarthes aura | NA | NA | mcp95 | 1311.4 |  |  |
| Catarthes aura | NA | NA | mcp95 | 1620.7 |  |  |
| Catarthes aura | NA | NA | mcp95 | 1801.1 |  |  |
| Catarthes aura | NA | NA | mcp95 | 34199.2 |  |  |
| Catarthes aura | NA | NA | mcp95 | 28027.1 |  |  |
| Catarthes aura | NA | NA | mcp95 | 28482.5 |  |  |
| Catarthes aura | NA | NA | mcp95 | 20555.1 |  |  |
| Catarthes aura | NA | NA | mcp95 | 17998.0 |  |  |
| Catarthes aura | NA | NA | mcp95 | 386.1 |  |  |
| Catarthes aura | NA | NA | mcp95 | 171.2 |  |  |
| Catarthes aura | NA | NA | mcp95 | 467.0 |  |  |
| Catarthes aura | NA | NA | mcp95 | 103.5 |  |  |
| Catarthes aura | NA | NA | mcp95 | 32.6 |  |  |
| Catarthes aura | NA | NA | mcp95 | 111.4 |  |  |
| Catarthes aura | NA | NA | mcp95 | 116.1 |  |  |
| Catarthes aura | NA | NA | mcp95 | 190.2 |  |  |
| Catarthes aura | NA | NA | mcp95 | 338.6 |  |  |
| Catarthes aura | NA | NA | mcp95 | 417.1 |  |  |
| Catarthes aura | NA | NA | mcp95 | 861.5 |  |  |
| Catarthes aura | NA | NA | mcp95 | 274.8 |  |  |
| Catarthes aura | NA | NA | mcp95 | 937.4 |  |  |
| Catarthes aura | NA | NA | mcp95 | 792.1 |  |  |
| Catarthes aura | NA | NA | mcp95 | 720.5 |  |  |
| Catarthes aura | NA | NA | mcp95 | 864.5 |  |  |
| Catarthes aura | NA | NA | mcp95 | 2930.1 |  |  |
| Catarthes aura | NA | NA | mcp95 | 654.1 |  |  |
| Catarthes aura | NA | NA | mcp95 | 5714.8 |  |  |
| Catarthes aura | NA | NA | mcp95 | 1023.8 |  |  |
| Catarthes aura | NA | NA | mcp95 | 67.7 |  |  |
| Catarthes aura | NA | NA | mcp95 | 5402.7 |  |  |
| Catarthes aura | NA | NA | mcp95 | 3488.1 |  |  |
| Catarthes aura | NA | NA | mcp95 | 3185.5 |  |  |
| Catarthes aura | NA | adult | kde95 | 4579 | 20273.5 | De Vault etal_2004 |
| Catarthes aura | NA | immature | kde95 | 5365 |  |  |
| Catarthes aura | NA | adult | kde95 | 6308 |  |  |
| Catarthes aura | NA | adult | kde95 | 4209 |  |  |
| Coragyps atratus | NA | adult | kde95 | 7788 |  |  |
| Coragyps atratus | NA | adult | kde95 | 41418 |  |  |
| Coragyps atratus | NA | adult | kde95 | 17128 |  |  |
| Coragyps atratus | NA | adult | kde95 | 9117 |  |  |
| Coragyps atratus | NA | adult | kde95 | 38699 |  |  |
| Coragyps atratus | NA | adult | kde95 | 40236 |  |  |
| Coragyps atratus | NA | adult | kde95 | 48161 |  |  |
| Vultur gryphus | male | adult | kde95 | 10580.5 | 16283.9 | Lambertucci etal_2014 |
| Vultur gryphus | female | adult | kde95 | 5736.6 |  |  |
| Vultur gryphus | female | adult | kde95 | 4944 |  |  |
| Vultur gryphus | female | adult | kde95 | 13468.5 |  |  |
| Vultur gryphus | female | adult | kde95 | 2670.7 |  |  |
| Vultur gryphus | female | adult | kde95 | 5709.3 |  |  |
| Vultur gryphus | female | adult | kde95 | 11663.8 |  |  |
| Vultur gryphus | male | adult | kde95 | 21378 |  |  |
| Vultur gryphus | male | adult | kde95 | 10672.2 |  |  |
| Vultur gryphus | male | adult | kde95 | 12865.2 |  |  |
| Vultur gryphus | female | adult | kde95 | 4240.3 |  |  |
| Vultur gryphus | male | adult | kde95 | 53254 |  |  |
| Vultur gryphus | male | adult | kde95 | 35978.1 |  |  |
| Vultur gryphus | female | adult | kde95 | 7191 |  |  |
| Vultur gryphus | male | adult | kde95 | 15166.9 |  |  |
| Vultur gryphus | male | adult | kde95 | 47874.2 |  |  |
| Vultur gryphus | female | adult | kde95 | 6511.2 |  |  |
| Vultur gryphus | male | adult | kde95 | 19301.3 |  |  |
| Vultur gryphus | female | adult | kde95 | 27231.8 |  |  |
| Vultur gryphus | female | adult | kde95 | 9239.7 |  |  |
| Vultur gryphus | female | adult | mcp100 | 14169 | 40396.5 | Pavez 2015 |
| Vultur gryphus | male | adult | mcp100 | 66624 |  |  |
| Vultur gryphus | male | adult | mcp100 | 243117 | 243117.0 | De Martino etal_2011 |
|  |  |  |  |  |  |  |
